# Performance of the ATRIA Bleeding Score in Predicting the Risk of In-Hospital Bleeding in Patients with ST-Elevation or Non-ST-Elevation Myocardial Infarction

**DOI:** 10.21470/1678-9741-2021-0027

**Published:** 2023

**Authors:** Fethi Yavuz, Mehmet Kaplan, Abdullah Yildirim, Omer Genc, Ramazan Asoglu, Abdulmecit Afsin, Yusuf Hosoglu, Salih Kilic

**Affiliations:** 1 Department of Cardiology, Adıyaman University Training and Research Hospital, Adıyaman, Turkey.; 2 Department of Cardiology, Gaziantep University, Gaziantep, Turkey.; 3 Department of Cardiology, Adana City Research & Training Hospital, Adana, Turkey.; 4 Department of Cardiology, Ağrı Training and Research Hospital, Ağrı, Turkey.

**Keywords:** Myocardial Infarction, Bleeding, Anticoagulants, SR Elevation Myocardial Infarctation, Atrial Fibrilation, Risk Factors, Risk Assessment

## Abstract

**Introduction:**

A clear assessment of the bleeding risk score in patients presenting with myocardial infarction (MI) is crucial because of its impact on prognosis. The Anticoagulation and Risk Factors in Atrial Fibrillation (ATRIA score is a validated risk score to predict bleeding risk in atrial fibrillation (AF), but its predictive value in predicting bleeding after percutaneous coronary intervention (PCI) in ST-segment elevation myocardial infarction (STEMI) or non-STEMI (NSTEMI) patients receiving antithrombotic therapy is unknown. Our aim was to investigate the predictive performance of the ATRIA bleeding score in STEMI and NSTEMI patients in comparison to the CRUSADE (Can Rapid risk stratification of Unstable angina patients Suppress ADverse outcomes with Early implementation of the American College of Cardiology/American Heart Association Guidelines) and ACUITY-HORIZONS (Acute Catheterization and Urgent Intervention Triage strategY-Harmonizing Outcomes with Revascularization and Stents in Acute Myocardial Infarction) bleeding scores.

**Methods:**

A total of 830 consecutive STEMI and NSTEMI patients who underwent PCI were evaluated retrospectively. The ATRIA, CRUSADE, and ACUITY-HORIZONS risk scores of the patients were calculated. Discrimination of the three risk models was evaluated using C-statistics.

**Results:**

Major bleeding occurred in 52 (6.3%) of 830 patients during hospitalization. Bleeding scores were significantly higher in the bleeding patients than in non-bleeding patients (all *P*<0.001). The discriminatory ability of the ATRIA, CRUSADE, and ACUITY-HORIZONS bleeding scores for bleeding events was similar (C-statistics 0.810, 0.832, and 0.909, respectively). The good predictive value of all three scores for predicting the risk of bleeding was observed in NSTEMI and STEMI patients as well (C-statistics: 0.820, 0.793, and 0.921 and 0.809, 0.854, and 0.905, respectively).

**Conclusion:**

This study demonstrated that the ATRIA bleeding score is a useful risk score for predicting major in-hospital bleeding in MI patients. This good predictive value was also present in STEMI and NSTEMI patient subgroups.

## INTRODUCTION

Advances in antithrombotic therapy, along with an early invasive strategy, have reduced the incidence of recurrent ischemic events and deaths in patients with myocardial infarction (MI). However, combined use of multiple pharmacotherapies including aspirin, P2Y12 receptor inhibitors, heparin plus glycoprotein IIb/IIIa inhibitors, direct thrombin inhibitors, and increased invasive procedures has been associated with an increased risk of bleeding^[1-2]^.

Hemorrhagic complications have emerged as an independent risk factor for mortality and morbidity in MI patients^[3]^. Bleeding is also associated with significantly prolonged hospital stay and increased utilization of healthcare resources, representing a source of excess expenditures^[4]^. Therefore, minimization of bleeding complications is an important goal in the management of ST-segment elevation myocardial infarction (STEMI) and non-STEMI (NSTEMI) patients. Increased awareness amongst clinicians of the importance of bleeding in these patients has led to the development of bleeding risk scores to guide the implementation of preventive strategies. Among these risk scores, the CRUSADE (standing for The Can Rapid Risk Stratification of Unstable Angina Patients Suppress Adverse Outcomes With Early Implementation of the ACC/AHA Guidelines) bleeding score is an established model that effectively predicts the risk of bleeding in patients presenting with NSTEMI^[5]^. The ACUITY-HORIZONS (standing for Acute Catheterization and Urgent Intervention Triage strategY-Harmonizing Outcomes with Revascularization and Stents in Acute Myocardial Infarction) bleeding risk score is another useful tool with demonstrated ability to predict bleeding in patients with acute coronary syndrome (ACS)^[6]^.

The algorithms of the CRUSADE and ACUITY-HORIZONS models are based on scoring systems that are too complex to be used in clinical practice. Although these bleeding scores generally have a satisfactory performance in acute in-hospital bleeding, there is a need for a simplified, easy-to-calculate scoring system for routine use. The Anticoagulation and Risk Factors in Atrial Fibrillation (ATRIA) bleeding score is a simple, easily calculated risk score which was originally developed to evaluate the risk of bleeding in patients with atrial fibrillation (AF) receiving long-term anticoagulant therapy^[7]^. In addition, there are studies in the literature that utilized the ATRIA bleeding score to predict bleeding risk associated with the use of antiplatelet drugs and oral anticoagulants in AF patients undergoing percutaneous coronary intervention (PCI)^[8]^. However, the predictive value of the ATRIA bleeding score for major bleeding in NSTEMI and STEMI patients without AF who are treated with antiplatelet drugs is unknown. Thus, we aimed to determine the predictive performance of the ATRIA bleeding score for major in-hospital hemorrhagic events in STEMI and NSTEMI patients in comparison to the CRUSADE and ACUITY-HORIZONS bleeding scores.

## METHODS

### Study Design

For the study, 830 consecutive patients with a definitive diagnosis of STEMI or NSTEMI who were admitted to the coronary intensive care unit of Adana City Hospital and underwent PCI between November 2018 and November 2019 were evaluated retrospectively. The exclusion criteria were patients undergoing coronary surgery, patients receiving conservative or fibrinolytic therapy, age under 18 and over 85 years, patients on chronic anticoagulant therapy for AF, prosthetic heart valve or any other indications, patients with missing data, pregnant patients, and patients whose coronary angiography images were unsuitable for analysis. The study was conducted in accordance with the principles set forth in the Declaration of Helsinki and ethics approval (date: 18/12/2019, approval number: 656) was obtained from the institutional review board.

Most of the registered patients received dual antiplatelet therapy (aspirin plus clopidogrel/ticagrelor/prasugrel) during their hospital stay unless they had bleeding. Coronary angiography and PCI were performed using the radial or femoral approach for arterial access. The lesions were treated with the use of contemporary interventional techniques. The choice of heparin therapy (unfractionated or low-molecular-weight heparin) was based on the recommendation of the individual patient’s attending cardiologist. For the study patients, the indication for initiation of treatment with glycoprotein IIb/IIIa inhibitors was also determined by the attending cardiologist. All demographic and clinical characteristics of the patients were recorded on admission. Laboratory data and detailed information on in-hospital pharmacological and interventional treatments were retrieved from the hospital’s electronic database. ATRIA, ACUITY-HORIZONS, and CRUSADE bleeding scores were calculated based on clinical and laboratory data collected at the time of admission using the original definitions of the respective trials.

### Clinical Endpoints and Definitions

STEMI was defined as typical chest pain for > 30 minutes but < 12 hours together with electrocardiographic change (ST-segment elevation of ≥ 1 mm in ≥ 2 contiguous leads, or new or presumably new left bundle branch block, or true posterior MI with ST depression of ≥ 1 mm in ≥ 2 contiguous anterior leads). NSTEMI was defined as typical chest pain for > 30 minutes and/or electrocardiographic change (ischemic ST-segment depression) accompanied by an elevated troponin-I level of ≥ 0.1 ng/ml^[9]^.

The ATRIA bleeding score was calculated using the following: anemia (hemoglobin < 13 g/dl in men and < 12 g/dl in women), renal impairment (estimated glomerular filtration rate [eGFR] < 30 or dialysis treatment), age (≥ 75 years), history of bleeding, and presence of hypertension^[7]^. The CRUSADE bleeding score was calculated using basal hematocrit, glomerular filtration rate (GFR), heart rate at presentation, systolic blood pressure at presentation, prior vascular disease, diabetes mellitus, symptoms of congestive heart failure at presentation, and sex^[5]^. The ACUITY-HORIZONS bleeding score was calculated using age, sex, serum creatinine concentration, white blood cell count, anemia, and troponin elevation^[6]^.

The primary endpoint was major bleeding events (type 3 or 5) during hospitalization as defined by the Bleeding Academic Research Consortium (BARC) criteria^[10]^:

Type 3a:

- Any transfusion with overt bleeding.
- Overt bleeding plus hemoglobin drop of ≥ 3 to 5 g/dL and corrected for transfusion (provided hemoglobin drop is related to bleeding).

Type 3b:

- Overt bleeding plus hemoglobin drop of ≥ 5 g/dL and corrected for transfusion (provided hemoglobin drop is related to bleed).
- Cardiac tamponade.
- Bleeding requiring surgical intervention for control (excluding dental/nasal/skin/hemorrhoid).
- Bleeding requiring intravenous vasoactive drugs.

Type 3c:

- Intracranial hemorrhage.
- Subcategories confirmed by autopsy or imaging or lumbar puncture.
- Intraocular bleed compromising vision.

Type 5:

- Fatal bleeding:

### Statistical Analysis

The study data were analyzed using the IBM Corp. Released 2017, IBM SPSS Statistics for Windows, Version 25.0, Armonk, NY: IBM Corp. The Kolmogorov-Smirnov test was used to check whether continuous variables followed a normal distribution. Normally distributed variables were expressed as mean ± standard deviation (or SD), while non-normally distributed variables were expressed as median with interquartile range (or IQR). The categorical variables were presented as percentages. Differences between the two groups were analyzed using the Student’s unpaired t-test or the Mann-Whitney U test for parameters with a normal or non-normal distribution. The frequencies of nominal variables were compared using the Fisher’s exact test or chi-square test. Pearson’s test was used for correlation analysis. The bleeding scores were classified into three risk strata. Thus, the patients were categorized as follows: low risk (0-3), intermediate risk (4), and high risk (5-10), using the ATRIA scores; low risk (≤ 30), intermediate risk (31-40), and high risk (> 40), based on the CRUSADE scores; and low risk (< 10), intermediate risk (10-14), or high risk (> 14), using the ACUITY-HORIZONS scores. The receiver-operating characteristic (ROC) curve analysis was used to determine the optimum cutoff levels for the ATRIA, CRUSADE, and ACUITY-HORIZONS bleeding risk scores that best predicted major bleeding. Discrimination was assessed by C-statistics, as the area under the curve (AUC) of each score for predicting major bleeding. A model with a C-statistic of 0.70 is generally considered to have acceptable discriminatory capacity^[11]^. All ROC comparisons were performed using the DeLong test. To determine independent predictors of bleeding, multivariate logistic regression analyses were performed.

## RESULTS

A total of 830 consecutive patients (mean age 61±10 years, 26.7% female) including 471 (57%) patients with STEMI and 359 (43%) patients with NSTEMI were retrospectively evaluated in the present study. During hospitalization, most of the patients underwent dual antiplatelet treatment plus full anticoagulation and only 10% received any of the glycoprotein IIb/IIIa inhibitors. None of the patients received bivalirudin. Major bleeding (BARC type 3 or 5 bleeding) occurred in 52 (6.3%) of 830 patients while staying in the hospital. The subgroup analysis showed that the incidence of major bleeding was 7.2% in STEMI patients and 5% in NSTEMI patients. Regarding the site of major bleeding, gastrointestinal bleeding was the most common, which occurred in 18 (35%) patients. Major bleeding at other sites was distributed as follows: genitourinary bleeding (n=13, 25%), vascular access hemorrhage (n=12, 23%), retroperitoneal bleeding (n=5, 10%), intracranial bleeding (n=2, 4%), and bleeding at multiple sites or a single undetermined site (n=2, 4%). Twenty-seven (52%) patients underwent an intervention directed at the bleeding site, including endoscopic intervention for 17 patients and surgical intervention for 10 patients. Bleeding patients had a mean hemoglobin drop of 4.2±1.2 mg/dl and received transfusion of a mean 2.2±1.7 units of red blood cell suspension.

Baseline demographic and clinical characteristics and laboratory data of the study patients are summarized in Table 1. When patients with or without major bleeding were compared with respect to demographic characteristics, hypertension, heart failure, coronary artery disease, prior PCI, alcoholism, renal failure, peptic ulcer, prior bleeding, prior beta-blocker use, and prior non-steroidal anti-inflammatory drug use were common among patients with major bleeding (*P*<0.05). Additionally, the mean age was higher and left ventricular ejection fraction was lower in patients in the major bleeding group (*P*=0.048 and *P*<0.001, respectively). There were no statistically significant differences between the study groups in other demographic and clinical characteristics (*P*>0.05). As for laboratory parameters, the major bleeding group showed significantly lower values for hemoglobin, hematocrit, GFR, albumin, higher levels of creatinine, urea, N-terminal pro-brain natriuretic peptide, and C-reactive protein (*P*<0.05). Other laboratory parameters were not significantly different between the two groups (*P*>0.05).

**Table 1:** Baseline demographics, clinical characteristics, and laboratory parameters of the study sample.

Parameters	All patients (n=830)	Bleeding		*P*-value
		Yes (n=52)	No (n=778)	
Age (years)	61.1±10.2	63.8±12.2	60.9±10	0.048
Female, n (%)	222 (26.7)	15 (28.8)	207 (26.6)	0.724
Body mass index, kg/m^2^	28.3±11.6	27.9±3.3	28.3±12	0.826
Systolic blood pressure (mmHg)	122±19.2	120±29.5	122±18.3	0.439
Diastolic blood pressure (mmHg)	77.1±15	75.8±23.4	77.2±14.3	0.538
Heart rate (beats/min)	81.5±12.9	84±15.4	81.2±12.7	0.142
Left ventricular ejection fraction, %	47.3±9.5	41.7±9.5	47.8±9.3	< 0.001
Medical history				
Hypertension, n (%)	352 (42.4)	33 (63.5)	319 (41)	0.002
Diabetes mellitus, n (%)	332 (40)	27 (51.9)	305 (39.2)	0.070
Dyslipidaemia, n (%)	167 (20.1)	7 (13.5)	160 (20.6)	0.216
Active smoking, n (%)	316 (38.1)	16 (30.8)	300 (38.6)	0.263
Heart failure, n (%)	76 (9.2)	16 (30.8)	60 (7.7)	< 0.001
Coronary artery disease, n (%)	229 (27.6)	21 (40.4)	208 (26.7)	0.033
Prior coronary bypass, n (%)	69 (8.3)	1 (1.9)	68 (8.7)	0.085
Prior PCI, n (%)	159 (19.2)	17 (32.7)	142 (18.3)	0.010
Alcoholism, n (%)	107 (12.9)	13 (25)	94 (12.1)	0.007
Stroke, n (%)	26 (3.1)	4 (7.7)	22 (2.8)	0.051
Renal failure, n (%)	31 (3.7)	15 (28.8)	16 (2.1)	< 0.001
Peptic ulcus, n (%)	69 (8.3)	14 (26.9)	55 (7.1)	< 0.001
Prior bleeding, n (%)	23 (2.8)	7 (13.5)	16 (2.1)	< 0.001
Prior aspirin, n (%)	194 (23.4)	19 (36.5)	175 (22.5)	0.210
Prior P2Y12 inhibitors, n (%)	52 (6.2)	3 (5.7)	39 (5)	0.676
Prior NSAİD, n (%)	154 (18.6)	18 (34.6)	136 (17.5)	0.020
Prior β-blocker, n (%)	192 (23.1)	25 (48.1)	167 (21.5)	< 0.001
Prior RAAS blockers, n (%)	272 (32.8)	23 (44.2)	249 (32)	0.069
Prior statins, n (%)	115 (13.9)	5 (9.6)	110 (14.1)	0.361
Laboratory parameters				
Fasting glucose level, mg/dL	168.1±83.2	186.2±81.6	166.9±83.2	0.106
White blood cells × 10^3^/µL	11.6±3.5	12±4.2	11.5±3.4	0.356
Hemoglobin, g/dL	13.7±1.9	12.3±2.0	13.9±1.8	< 0.001
Hematocrit, %	39.9±5.1	36±5.2	40.1±5.0	< 0.001
Platelets, ×10^3^/mL	264.9±73.2	260±93.3	265±71.7	0.662
Creatinine, mg/dL	0.8±(0.7-1.0)	1.2(0.8-1.7)	0.8 (0.7-0.9)	< 0.001
Urea, mg/dL	36.6±14.8	54.4±27.1	35.5±12.7	< 0.001
eGFR, mL/min	89.4±21.3	64.6±31.3	91±19.4	< 0.001
Sodium, mmol/dL	137.4±2.8	137.1±3.4	137.4±2.8	0.495
Potassium, mmol/dL	4.3±0.5	4.4±0.5	4.3±0.5	0.151
Serum uric acid, mg/dL	5.7±1.6	5.9±1.7	5.7±1.6	0.345
Alanine transaminase, U/L	24 (18-33)	27 (18-38)	24 (17-33)	0.220
Aspartate aminotransferase, U/L	32 (23-49)	33 (26.2-49)	32 (23-49.3)	0.450
Albumin, mg/dL	3.8±0.4	3.6±0.4	3.8±0.4	0.002
NT-proBNP, pg/mL	775 (256-2230)	2700 (1253-8070)	698 (256-2020)	< 0.001
C-reactive protein, mg/L	5.1 (2.4-11.1)	7.8 (4.0-12.4)	5 (2.4-10.9)	0.028
International normalised ratio	1±0.13	1.02±0.13	1.0±0.13	0.285

eGFR=estimated glomerular filtration rate; NSAID=non-steroidal anti-inflammatory drugs; NT-proBNP=N-terminal pro-B-type natriuretic peptide; PCI=percutaneous coronary intervention; RAAS=renin-angiotensin-aldosterone system

Baseline clinical characteristics, in-hospital treatment, and bleeding scores of the study population are summarized in Table 2. In-hospital P2Y12 inhibitors switch and anticoagulant switch as well as the use of glycoprotein IIb/IIIa inhibitors were significantly more common in the major bleeding group (*P*=0.021, *P*=0.002, and *P*<0.001, respectively). The risk of major bleeding increased significantly with increase in Killip class (*P*<0.001). The mean ATRIA, CRUSADE, and ACUITY-HORIZONS bleeding risk scores of the study sample were 1.5±1.7, 26.4±11.1, and 13.2±6.7 respectively. When the bleeding scores of the two groups were analyzed, the major bleeding group showed significantly higher mean ATRIA, CRUSADE, and ACUITY-HORIZONS bleeding scores (*P*<0.001). Patients with bleeding had longer hospitalisation time than non-bleeding patients (*P*<0.001). There was no significant difference between the two groups in terms of culprit coronary vessel and clinical presentation (*P*>0.05).

**Table 2:** Baseline clinical characteristics, in-hospital treatment, and bleeding scores of the study sample.

Parameters	All patients (n=830)	Bleeding		*P*-value
		Yes (n=52)	No (n=778)	
**Clinical presentation**				
NSTEMI, n (%)	359 (43,2)	18 (34.6)	341 (43,8)	0.194
STEMI, n (%)	471 (56.8)	34 (65.4)	437 (56,2)	
**Arterial access site**				
Femoral, n (%)	707 (85.2)	49 (94.2)	658 (84.6)	0.058
Radial, n (%)	123 (14.8)	3 (5.8)	120 (15.4)	
**Culprit vessel**				
Left anterior descending, n (%)	362 (43.6)	26 (50)	336 (43.2)	> 0.05
Circumflex artery, n (%)	182 (21.9)	15 (28.8)	167 (21.5)	
Right coronary artery, n (%)	257 (31)	10 (19.3)	247 (31.7)	
Others, n (%)	29 (3.5)	1 (1.9)	28 (3.6)	
**Killip class**				
Class 1, n (%)	598 (72.0)	11 (21.2)	587 (75.5)	< 0.001
Class 2, n (%)	139 (16.7)	16 (30.8)	123 (15.8)	
Class 3, n (%)	48 (5.8)	9 (17.3)	39 (5.0)	
Class 4, n (%)	45 (5.5)	16 (30.7)	29 (3.7)	
In-hospital time, days	3.9±1.8	4.9±1.9	3.8±1.7	< 0.001
**In-hospital treatment**				
Aspirin, n (%)	817 (98.4)	50 (96.2)	767 (98.6)	0.171
P2Y12 inhibitors switch, n (%)	94 (11.3)	11 (21.2)	83 (10.7)	0.021
Anticoagulant switch, n (%)	67 (8.1)	10 (19.2)	57 (7.3)	0.002
Glycoprotein IIb/IIIa inhibitors, n (%)	85 (10.3)	18 (34.6)	67 (8.6)	< 0.001
**Bleeding risk score**				
ATRIA score	1.5±1.7	3.76±2.2	1.33±1.6	< 0.001
CRUSADE score	26.4±11.1	41.9±13.1	25.4±10.2	< 0.001
ACUITY-HORİZONS score	13.2±6.7	23.7±5.2	12.5±6.2	< 0.001

ACUITY-HORIZONS=Acute Catheterization and Urgent Intervention Triage StrategY-Harmonizing Outcomes with Revascularization and Stents in Acute Myocardial Infarction; ATRIA=Anticoagulation and Risk Factors in Atrial Fibrillation; CRUSADE=The Can Rapid Risk Stratification of Unstable Angina Patients Suppress Adverse Outcomes With Early Implementation of the ACC/AHA Guidelines; NSTEMI=non-ST-segment elevation myocardial infarction; STEMI=ST-segment elevation myocardial infarction

Table 3 shows subclassification of the bleeding scores into low, intermediate, and high-risk categories and their correlation with major bleeding. While the patients showed a more homogeneous distribution for the ACUITY-HORIZONS scores, there was a non-homogeneous distribution of patients for the CRUSADE and ATRIA scores, with the greatest number of patients in the low-risk category. On all three bleeding scores, patients with major bleeding were identified at high risk for bleeding. The risk of major bleeding increased significantly when moving from the low-risk group to the high-risk group in all bleeding scores (*P*<0.001).

**Table 3:** ATRIA, CRUSADE, and ACUITY HORIZONS bleeding scores subcategorized to low, intermediate, and high risk.

Bleeding scores	All patients (n=830)	Bleeding		*P*-value
		Yes (n=52)	No (n=778)	
**ATRIA score**				
Low risk (0-3)	697	19 (2.7%)	678 (97.3%)	< 0.001
Intermediate risk (4)	77	9 (11.7%)	68 (88.3%)	
High risk (5-10)	56	24 (42.9%)	32 (57.1%)	
**CRUSADE score**				
Low risk (≤ 30)	569	13 (2.3%)	556 (97.7%)	< 0.001
Intermediate risk (31-40)	170	11 (6.5%)	159 (93.5%)	
High risk (> 40)	91	28 (30.8%)	63 (69.2%)	
**ACUITY-HORIZONS score**				
Low risk (< 10)	281	0 (0%)	281 (100%)	< 0.001
Intermediate risk (10-14)	229	3 (1.3%)	226 (98.7%)	
High risk (> 14)	320	49 (15.3%)	271 (84.7%)	

ACUITY-HORIZONS=Acute Catheterization and Urgent Intervention Triage StrategY-Harmonizing Outcomes with Revascularization and Stents in Acute Myocardial Infarction; ATRIA=Anticoagulation and Risk Factors in Atrial Fibrillation; CRUSADE=The Can Rapid Risk Stratification of Unstable Angina Patients Suppress Adverse Outcomes With Early Implementation of the ACC/AHA Guidelines

On Pearson’s correlation analysis comparing the bleeding scores with each other, the ATRIA bleeding score showed statistically significantly positive correlations with CRUSADE and ACUITY-HORIZONS bleeding scores (r=0.597, *P*<0.001 and r=0.641, *P*<0.001, respectively). In addition, a significant positive correlation was observed between the CRUSADE score and the ACUITY-HORIZONS score (r=0.659, *P*<0.001).

**Fig. 1:**
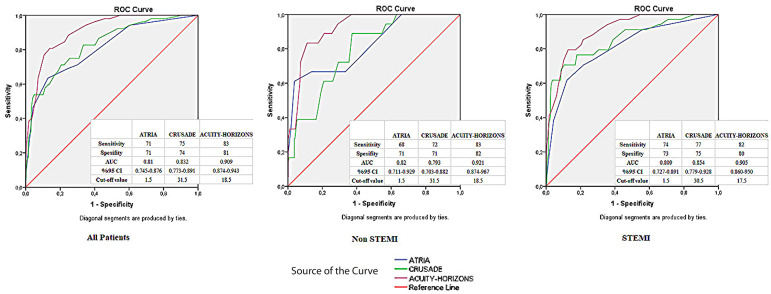
Receiver-operating characteristic (ROC) curves of major bleeding according to the ATRIA, CRUSADE, and ACUITY HORIZONS scores in the entire cohort and STEMI and non-STEMI subgroups. ACUITY-HORIZONS=Acute Catheterization and Urgent Intervention Triage strategY-Harmonizing Outcomes with Revascularization and Stents in Acute Myocardial Infarction; ATRIA=Anticoagulation and Risk Factors in Atrial Fibrillation; AUC=area under the curve; CI=confidence interval; CRUSADE=The Can Rapid Risk Stratification of Unstable Angina Patients Suppress Adverse Outcomes With Early Implementation of the ACC/AHA Guidelines.

The ROC curves of major bleeding are shown in Figure 1 for the study sample and STEMI and NSTEMI subgroups. For the prediction of major bleeding in all patients, the cutoff value of > 1.5 for ATRIA score had a 71% sensitivity and a 71% specificity in the ROC curve analysis. For the prediction of major bleeding in the NSTEMI subgroup, the cutoff value of > 1.5 for ATRIA score had a 68% sensitivity and a 71% specificity. For the prediction of major bleeding in the STEMI subgroup, the cutoff value of > 1.5 for ATRIA score had a 74% sensitivity and a 73% specificity.

The discriminatory ability of the ATRIA, CRUSADE, and ACUITY-HORIZONS bleeding scores for bleeding events was similar (C-statistics and 95% confidence interval [CI]: 0.810 [0.745-0.876], 0.832 [0.773-0.891], and 0.909 [0.874-0.943], respectively). All three bleeding scores showed a good predictive value for predicting major bleeding among non-STEMI patients (C-statistics and 95% CI: 0.820 [0.711-0.929], 0.793 [0.703-0.882], and 0.921 [0.874-0.967], respectively) and STEMI patients (C-statistics and 95% CI: 0.809 [0.727-0.891], 0.854 [0.779-0.928], and 0.905 [0.860-0.950], respectively). We performed a pairwise comparison of ROC curves for the predictive value of ATRIA score with regards to bleeding which was similar to CRUSADE score, but ACUITY-HORIZONS score was superior to ATRIA score and CRUSADE score in all study population (by DeLong method, AUC_ATRIA_ *vs.* AUC_CRUSADE_ z-test=0.742, *P*=0.458; AUC_ACUITY-HORIZONS_ *vs.* AUC_ATRIA_ z-test=3.116, *P*=0.002; AUC_ACUITY-HORIZONS_*vs.* AUC_CRUSADE_ z-test=2.598, *P*=0.009). In the subgroup analyses, the predictive value of ATRIA score with regards to bleeding was similar to CRUSADE score, but ACUITY-HORIZONS score was superior to ATRIA score in STEMI patients (AUC_ATRIA_ *vs.* AUC_CRUSADE_ z-test=1.147, *P*=0.251; AUC_ACUITY-HORIZONS_ *vs.* AUC_ATRIA_ z-test=2.480, *P*=0.013; AUC_ACUITY-HORIZONS_ *vs.* AUC_CRUSADE_ z-test=1.378, *P*=0.168). In the NSTEMI patients, the predictive value of ATRIA score was similar to ACUITY HORIZONS and CRUSADE score (AUC_ATRIA_ *vs.* AUC_CRUSADE_ z-test=0.732, *P*=0.464; AUC_ACUITY-HORIZONS_ *vs.* AUC_ATRIA_ z-test=1.806, *P*=0.071; AUC_ACUITY-HORIZONS_ *vs.* AUC_CRUSADE_ z-test=2.821, *P*=0.005).

Multivariate regression analysis results are summarized in Table 4. In the multivariate logistic regression analysis, non-steroidal anti-inflammatory medication history (xv=0.022), prior bleeding (*P*=0.001), left ventricular ejection fraction (v=0.005), hemoglobin (*P*=0.022), eGFR (*P*=0.002), arterial access site (*P*=0.05), and glycoprotein IIb/IIIa inhibitors use (*P*<0.001) were independent predictors of bleeding in all study population.

**Table 4:** Multivariate logistic regression analysis for predictors of bleeding events.

Analysis	Multivariate	
Variables	*P*-value	OR (95% CI)
Prior non-steroidal anti-inflammatory drug use	0.022	0.363 (0.153-0.865)
Prior bleeding	0.001	0.093 (0.023-0.382)
Left ventricular ejection fraction	0.005	0.936 (0.893-0.980)
Hemoglobin	0,022	0.762 (0.604-0.961)
Estimated glomerular filtration rate	0.002	0.974 (0.957-0.990)
Arterial access site	0.050	0.200 (0.040-1.003)
Glycoprotein IIb/IIIa inhibitors use	< 0.001	4.710 (2.049-10.824)

CI=confidence interval; OR=odds ratio

## DISCUSSION

This study demonstrated that the ATRIA bleeding score is a useful risk score for the prediction of in-hospital major bleeding in NSTEMI and STEMI patients. Compared to the CRUSADE and ACUITY-HORIZONS scores, two well-established bleeding scores to predict bleeding events, ATRIA bleeding score is simpler to calculate and showed a similar predictive value to estimate the risk of major bleeding in the present study (C-statistics ATRIA: 0.810, CRUSADE: 0.832, and ACUITY-HORIZONS: 0.909). This also applied for the subgroups of STEMI and NSTEMI patients.

Until recently, bleeding was considered as an inevitable complication of the treatment of STEMI and NSTEMI patients. Some increase in the bleeding risk seemed acceptable provided that antiplatelet and anticoagulant agents reduced the incidence of recurrent ischemic events. However, the studies have increasingly shown that the bleeding episode itself is associated with adverse outcomes including MI and death^[3,12]^. For example, in a study retrospectively examining the follow-up of 10,974 patients who underwent PCI, Kinnaird et al.^[12]^ found a significant increase in major adverse cardiac events (death, recurrent MI, and revascularization) with increased bleeding severity. Similarly, Eikelboom et al.^[13]^ investigated the impact of bleeding on prognosis in 34,146 NSTEMI patients and reported a significant association between major bleeding and 30-day mortality. These studies demonstrate the relationship between bleeding and other adverse outcomes and suggest that reduction of bleeding is an attractive therapeutic goal that can improve survival in NSTEMI and STEMI patients, provided that ischemic events are also reduced.

Our study population consisted of NSTEMI and STEMI patients. The incidence of major bleeding was 6.3% in the current study. From the ACTION Registry-GWTG (standing for Acute Coronary Treatment and Intervention Outcomes Network Registry-Get with the Guidelines) database, the rate of in-hospital major bleeding in the overall population was 10.8%^[14]^. In a study involving 17,421 ACS patients, Mehran et al.^[6]^ reported major bleeding within 30 days at an incidence of 4.3%. Abu-Assi et al.^[15]^ found a major bleeding rate of 9.5% among patients with NSTEMI. In a study by Correia et al.^[16]^, the incidence of major bleeding was 6% in ACS patients. A low rate (2.4%) of major bleeding in STEMI patients at one year was reported by Liu et al.^[17]^. However, radial artery access was used for interventions in 92% of the patients enrolled in that study. As known from the results of randomized trials, access site complications can be reduced by 78% with the use of the radial approach. In a study by Ariza-Solé et al.^[18]^, the incidence of major in-hospital bleeding was 3.1% in STEMI patients. Radial access was used for interventions in 58.2% of the patients and dual antiplatelet therapy consisting of aspirin and clopidogrel were administered in that study. Furthermore, one-fifth of the patients were receiving bivalirudin. In the current study, the patients received a combination of aspirin and clopidogrel/prasugrel/ticagrelor as dual antiplatelet therapy as recommended by current guidelines. Glycoprotein IIb/IIIa inhibitors were used in 10.3% of the patients. However, none of our patients used bivalirudin because this drug is not available in Turkey. The wide variation in the reported incidence of bleeding in the literature may be attributed to a number of factors including differences in patient characteristics, differences in concurrent treatments, and differences in the timing of event reporting, definitions of bleeding, and interventional procedures among studies. Due to these limitations and the variability in the definitions used, the rates of major bleeding reported by published studies vary between 1% and 10%^[19,20]^.

In the current study, major bleeding occurred at a higher incidence in the STEMI subgroup than in the NSTEMI subgroup (7.2% *vs.* 5%, respectively). A higher incidence of major bleeding in the STEMI subgroup compared to the NSTEMI subgroup was also observed in the study by Mehran et al.^[6]^ (6.2% *vs.* 4.4%, respectively). Similarly, major bleeding was found in 11.8% of the STEMI patients and 10.2% of NSTEMI patients in a subgroup analysis of the ACTION Registry-GWTG database^[14]^. The increased rate of bleeding in STEMI patients compared with NSTEMI patients might reflect the urgency of care provided, more frequent use of large arterial sheaths, unadjusted patient comorbidities, and the more frequent use of a loading dose of P2Y12 inhibitors^[21]^.

Despite differences among studies in the incidence of bleeding and the definitions used for major bleeding, advanced age, female sex, low body weight, use of invasive procedures, comorbidities such as hypertension, multiple pharmacotherapies, and renal failure have been consistently identified in several studies as strong predictors of ACS and bleeding complications of PCI. Age, renal insufficiency, and use of invasive procedures stand out as the most important risk factors for bleeding irrespective of the antithrombotic strategy^[22-24]^. Advancing age is a strong risk factor for bleeding. In a study analyzing the data from the Global Registry of Acute Coronary Events (or GRACE) encompassing the entire ACS spectrum, the likelihood of experiencing a major bleeding prior to discharge increased by about 30% per each decade^[23]^. Contemporary ACS registries have shown that patients with renal failure have a 50% increased estimated risk of in-hospital major bleeding. This increase is thought to be mediated by a number of mechanisms including platelet dysfunction, endothelial cell dysregulation, activation of the fibrinolytic system, and overdosage or accumulation of antithrombotic drugs^[24,25]^. Consistently, older age and renal failure were statistically more common among bleeding patients in the present study. However, in contrast to the literature, no statistical association was found between bleeding and sex or body mass index in our study. Moreover, all of our patients underwent invasive procedures.

The ATRIA bleeding score was developed to predict bleeding related to oral anticoagulation therapy and clinical outcomes in patients with AF^[7]^. Recent studies have investigated the role of the ATRIA bleeding score in predicting the risk of bleeding in ACS patients with AF receiving anticoagulant therapy. Kiviniemi et al.^[8]^ reported a major bleeding rate of 10.4% at one-year follow-up among AF patients who received oral anticoagulant therapy and dual antiplatelet drugs after PCI. Unlike our study, patients with stable or unstable angina pectoris were also included in that study. Another difference is that while there was a one-year follow-up in the study by Kiviniemi et al.^[8]^, our study investigated in-hospital major bleeding events. The authors of that study concluded that the ATRIA score and other bleeding scores developed for AF had no predictive value in predicting bleeding complications. However, in our study, the ATRIA bleeding score showed a good predictive ability in predicting in-hospital major bleeding. Although AF patients and patients receiving oral anticoagulants for any indication were excluded from the current study, all of the study patients underwent PCI and received intravenous anticoagulant (unfractionated or low-molecular-weight heparin) therapy.

STEMI and NSTEMI create a high-risk clinical setting for bleeding and require more aggressive pharmacological therapy and invasive strategies that are associated with increased risk of bleeding complications. Given the strong correlation between bleeding and subsequent mortality, bleeding prediction models are important for risk stratification and decisions regarding treatment. The CRUSADE and ACUITY-HORIZONS bleeding scores have proven accuracy in predicting bleeding in ACS patients^[5,6]^. However, since the algorithms of these scoring systems are complex and difficult to calculate, a simpler bleeding score will obviously be more convenient for clinicians. Although the ATRIA bleeding score has not been developed specifically for STEMI and NSTEMI, it is simple to calculate and consists of five clinical variables (namely, anemia, renal failure, age [≥ 75 years], prior bleeding, and hypertension) which have been independently shown to be associated with bleeding^[23,24]^. The good predictive ability of the ATRIA bleeding score in predicting major bleeding in the current cohort of STEMI and NSTEMI patients may be explained with the established association of the aforementioned parameters with bleeding.

Although efficacy as it has been classically described (death, MI, revascularization) should still be the primary focus in the treatment of MI patients, there is emerging evidence that the traditional safety endpoint of bleeding affects at least two components of the composite efficacy (death and MI)^[12,13]^. It is important to note that the inclusion of bleeding in the efficacy endpoint does not mean that a sacrifice should be made with regard to death or MI. Rather, it means that one must be willing to accept a potentially small reduction in efficacy for a large benefit in safety. Identification of patients with a greater tendency for bleeding may lead to improved care of NSTEMI and STEMI patients by prompting clinicians to make rational treatment decisions, to carefully dose antithrombotic drugs, and to choose invasive strategies to optimize patient-centered care. Our study showed that the ATRIA bleeding score can be used in the risk stratification for bleeding in STEMI and NSTEMI patients.

### Limitations

The current study has a number of limitations. Firstly, the sample size was small and, consequently, the number of events was low. Secondly, this study was designed as an analysis of the data from a single center, which were retrospectively collected from a clinical registry, and this may limit the generalizability of our findings. Thirdly, we did not compare the three scores using their main bleeding definitions but instead used the BARC criteria. Another limitation is the exclusion of patients with unstable angina pectoris. However, although our study sample was relatively small, it represents a well-balanced, contemporary ACS population with almost equal numbers of STEMI and NSTEMI cases.

## CONCLUSION

The findings of the present study show that, as a practical and convenient scoring system, the ATRIA bleeding score is useful in predicting in-hospital major bleeding in STEMI and NSTEMI patients without AF. This good predictive value was observed in the STEMI and NSTEMI subgroups as well. Compared to the CRUSADE and ACUITY-HORIZONS scores, the ATRIA bleeding score was easier to calculate and had similar accuracy for risk assessment.
